# The microbiota composition of the offspring of patients with gestational diabetes mellitus (GDM)

**DOI:** 10.1371/journal.pone.0226545

**Published:** 2019-12-16

**Authors:** Valentina Ponzo, Ilario Ferrocino, Adriana Zarovska, Maria Bernadette Amenta, Filomena Leone, Clara Monzeglio, Rosalba Rosato, Marianna Pellegrini, Roberto Gambino, Maurizio Cassader, Ezio Ghigo, Luca Cocolin, Simona Bo

**Affiliations:** 1 Department of Medical Sciences, University of Turin, Turin, Italy; 2 Department of Agricultural, Forestry, and Food Science, University of Turin, Turin, Italy; 3 Clinical Nutrition Unit, S. Anna Hospital, Città della Salute e della Scienza, Turin, Italy; 4 Gynecology and Obstetrics Unit, S. Anna Hospital, Città della Salute e della Scienza, Turin, Italy; 5 Department of Psychology, University of Turin, Turin, Italy; University of Illinois, UNITED STATES

## Abstract

The microbiota composition of the offspring of women with gestational diabetes mellitus (GDM), a common pregnancy complication, is still little known. We investigated whether the GDM offspring gut microbiota composition is associated with the maternal nutritional habits, metabolic variables or pregnancy outcomes. Furthermore, we compared the GDM offspring microbiota to the microbiota of normoglycemic-mother offspring. Fecal samples of 29 GDM infants were collected during the first week of life and assessed by 16S amplicon-based sequencing. The offspring’s microbiota showed significantly lower α-diversity than the corresponding mothers. Earlier maternal nutritional habits were more strongly associated with the offspring microbiota (maternal oligosaccharide positively with infant *Ruminococcus*, maternal saturated fat intake inversely with infant *Rikenellaceae* and *Ruminococcus*) than last-trimester maternal habits. Principal coordinate analysis showed a separation of the infant microbiota according to the type of feeding (breastfeeding *vs* formula-feeding), displaying in breast-fed infants a higher abundance of *Bifidobacterium*. A few *Bacteroides* and *Blautia* oligotypes were shared by the GDM mothers and their offspring, suggesting a maternal microbial imprinting. Finally, GDM infants showed higher relative abundance of pro-inflammatory taxa than infants from healthy women. In conclusion, many maternal conditions impact on the microbiota composition of GDM offspring whose microbiota showed increased abundance of pro-inflammatory taxa.

## Introduction

It is well known that the maternal environment affects the offspring health. The newborn gut microbiota is strongly influenced by maternal health and pregnancy conditions and participates in the development programming of the newborns [1–3]. Early disruption of the infant microbiota has been associated with many inflammatory, immune-mediated, allergic, and dysmetabolic diseases in later life [1,4–5]. Children’s obesity, non-alcoholic hepatic liver diseases, aberrant cardiac growth are, among others, the conditions that have been associated with maternal/newborn dysbiosis [1,6–7]. Nevertheless, uncertainty still exists about the microbiota offspring colonization, and both the modality and the timing of microbial exposure for the fetus/newborn are controversial [8–11].

Gestational diabetes mellitus (GDM), the most frequent complication of pregnancy, has been associated with increased risk for the offspring of developing dysmetabolic diseases, such as obesity and diabetes mellitus [12]. Many prenatal and postnatal factors could determine an increased metabolic risk, such as epigenetic changes induced by in-utero exposure to maternal hyperglycemia, impaired secretion of fetal hormones (insulin and leptin), or fetal “mal-programming” of hypothalamic neuropeptidergic neurons, leading to hyperphagia [13–14]. GDM was found to be associated with specific changes in the gut microbiota composition [15–19]. The altered microbiome may be a bystander of the underlying metabolic dysregulation that underpins the pathogenesis of gestational hyperglycemia, as well as the consequence of the increased adiposity frequently co-existing in GDM patients [20–21]. However, the finding that a different microbial pattern precedes the onset of GDM leads to the hypothesis that microbiota alterations might have a role in the pathogenesis of GDM [22–23]. Even more important is the reporting that specific taxa associated with GDM can be transmitted to the offspring and differentiate their gut microbiota from that of the offspring of normoglycemic women [16,24–25].

The possibility of early modulation of the offspring gut microbiota by acting on specific maternal factors and/or characteristics is of potential great interest. Nevertheless, only few data are available about the associations between maternal characteristics and newborn microbiota pattern. Maternal fasting glucose concentrations were correlated positively with the relative abundance of phylum Actinobacteria and genus *Acinetobacter*, and negatively with the phylum Bacteroidetes and the genus *Prevotella* [26]. We hypothesized that maternal dietary habits and metabolic characteristics may impact on the offspring gut microbiota.

Aims of the present observational study were therefore evaluating whether maternal nutritional habits and/or metabolic variables and pregnancy outcomes of GDM patients are associated with the gut microbiota composition of their offspring. Furthermore, the gut microbiota composition of these infants was compared to the gut microbiota of the offspring of healthy normoglycemic women to evaluate possible differences between the two cohorts of newborns.

## Materials and methods

### Patients recruitment

All pregnant women routinely performed an oral glucose tolerance test at 24–28 gestational weeks at the “Città della Salute e della Scienza” Hospital of Turin. The first 50 consecutive subjects who met both the criteria for GDM according to international guidelines (fasting plasma glucose ≥92 mg/dL and/or 1h post-test glycemia ≥180 mg/dL and/or 2h post-test glycemia ≥153 mg/dL) and the inclusion criteria (see below) were enrolled [17]. Out of them, 41 women participated in an observational study aiming to evaluate the changes in gut microbiota composition across pregnancy [17]. The fecal samples of their offspring were collected in the first week of life. Indeed, as the mode of collection and/or storage of the offspring stool samples by 12 mothers resulted inappropriate, data of 29 infants (70.7%) could be analyzed only (S1 Table). Inclusion criteria were: GDM diagnosed by a 75g oral glucose tolerance test (OGTT) between 24–28 weeks gestational age and European origin with both parents born in Europe. Women with twin pregnancy, any pathological conditions before or during pregnancy (known diabetes mellitus, hypertension, cardiovascular, pulmonary, autoimmune, joint, liver or kidney diseases, thyroid dysfunction, cancer, any other disease/condition), no compliance to the study protocol, on prebiotics/probiotics, antibiotics or any drug during pregnancy were excluded. All the GDM patients received dietary counselling and the recommendation of performing 30-min daily moderate exercise (i.e. brisk walking) and were instructed to self-monitor finger-prick capillary blood glucose at least 4 times per day. Participants completed a 3-day food record (2 weekdays and 1 weekend day) and the Minnesota-Leisure-Time-Physical Activity Questionnaire at enrolment and at the study end [17]. Detailed information on how to record the food and drink consumed by using common household measures was provided to all participants. Two dieticians checked all questionnaires for completeness, internal coherence and plausibility [17].

Patients were considered to be compliant to the given dietary recommendations in the presence of all the following criteria: at least 20 g/day fiber consumption (or increasing fiber intake more than 50% than enrolment), sugar reduction to less than 10% of total energy and abolition of alcohol intake. Only 14 out of 41 (34.1%) of the patients were compliant to dietary recommendations. Finally, all mothers were suggested to breastfeed their children. However, only 10 of them followed this suggestion. All the others declared to have used formulas without added probiotic/prebiotic compounds.

In order to compare our offspring to infants from normoglycemic women, we have used the recently released 16S rRNA gene sequence from 19 Italian infants collected in the first week of life [27] (NCBI SRA, under BioProject ID PRJNA378341). Infants from healthy women showed the following characteristic: (i) born from vaginal delivery, (ii) breastfed, (iii) no use of antibiotic/probiotic during pregnancy [27]. We used as comparison the results of the 4^th^ day of life.

### Ethical aspects

The present study conforms to the principles outlined in the Declaration of Helsinki. The study protocol was approved by the Ethics Committee of the “Città della Salute e della Scienza” hospital of Turin (approval 707/2016). All patients provided written informed consent prior to participation in the study protocol.

### Data and samples collections

Anthropometric values, 3-days food record questionnaires, fasting blood samples (for the determination of metabolic variables and C-reactive protein levels) and stool samples of the mothers were collected both at the time of GDM diagnosis, at 24–28 weeks of gestational age, and at 38 weeks, or before delivery, in the case of preterm delivery [17]. Fecal samples of the offspring were collected between the 3^rd^ and the 5^th^ day of life, after meconium expulsion. Data relative to the type of delivery, birth weight, gestational age and the type of feeding were extracted from medical records.

Insulin therapy was added to diet when fasting blood glucose levels were consistently ≥90 mg/dL, 1-hour levels consistently ≥130 mg/dL, or 2-hour levels ≥120 mg/dL, according to guidelines [28].

Mothers were instructed to collect the stool samples and all materials were provided in a convenient, refrigerated, specimen collection kit (VWR, Milan, Italy) [17]. The fecal samples were transferred to the sterile sampling containers using a polypropylene spoon (3 spoons of about 10 g) and immediately stored at 4 °C. The specimens were transported to the laboratory within 12 hours of collection at a refrigerated temperature. Containers were immediately stored at −80 °C for DNA extraction. No storage medium was used [17].

In the case of infants from normoglycemic mothers, fecal samples have been reported to be collected from diapers using standard sterile collection tubes [27].

### Fecal DNA extraction and sequencing

Total DNA from the feces collected was extracted using the RNeasy Power Microbiome KIT (Qiagen, Milan, Italy) following the manufacturer’s instructions. One microliter of RN*ase* (Illumina Inc. San Diego. CA) was added to digest RNA in the DNA samples, with an incubation of 1 h at 37 °C. DNA was quantified using the QUBIT dsDNA Assay kit (Life Technologies, Milan, Italy) and standardized at 5 ng/μL. DNA was used as a template in a PCR reaction in order to amplify the V3-V4 region of the 16S rRNA gene [29]. Library preparation and sequencing (2X250bp) was performed as already reported [17].

### Bioinformatics analyses

Paired-end reads were first assembled using FLASH software and quality filtering using QIIME 1.9.0 software [30] and the pipeline already reported [17]. OTUs picked at 97% of similarity were rarefied to the lowest number of sequences per sample. The OTU table obtained through QIIME displays the higher taxonomy resolution that was reached; when the taxonomy assignment was not able to reach the genus, family name was displayed.

In order to identify sub-OTUs populations in our offspring cohort, reads assigned to genera *Blautia*, *Bacteroides* and *Bifidobacterium* were extracted and entropy analysis and oligotyping were carried out [31] since these were the only OTUs showing higher level of entropy able to obtain sub-OTUs. After the first round of oligotyping, high entropy positions were chosen (-C option): 8, 12, 113, 223, 224, 247, 432, 433 for *Blautia*; position 8, 12, 40, 74, 75, 116, 117, 124, 129, 130, 390, 397, 452 and 453 were chosen for *Bacteroides*; while position 8, 12, 14, 81, 114, 115, 158, 159, 160, 328, 329, 389, 437, 438, 439, 440, 441, 442, 443 and 444 were chosen for *Bifidobacterium*. In order to reduce the noise, each oligotype should appear in at least 10 samples, occur in more than 1.0% of the reads for at least ten samples, represent a minimum of 750 reads in all samples combined, and have a most abundant unique sequence with a minimum abundance of 50. A cladogram of representative sequences was generated using the ANVIOs software [32].

### Statistical analyses

Gut microbiota α-diversity was calculated by the diversity function of the vegan package [33] in R environment (http://www.r-project.org). ADONIS and ANOSIM statistical test were used to detect significant differences in the overall microbial community by using the unweighted UniFrac distance matrices or OTU table. Offspring variables were compared with maternal variables by paired-sample t-test, or Wilcoxon matched pairs test, as appropriate. Differences in infant gut microbiota between mothers who were compliant or not to the dietary recommendations, or between the GDM offspring and healthy-women offspring were calculated by t-Student test or Mann-Whitney test. Pairwise Spearman’s non-parametric correlations were used to study the relationships between the infant relative abundance of microbial taxa and maternal dietary and metabolic variables. The degree of agreement (concordance) among maternal oligotypes at enrolment, maternal oligotypes at the study-end and infant oligotypes was assessed by the Friedman ANOVA & Kendall’s test.

Multiple regression analyses were performed to evaluate the associations between infant microbial taxa abundance (dependent variable) and maternal nutrient intakes or laboratory variables, after adjusting for gestational weight change, breastfeeding and Cesarean section (Statistica, ver. 7.0; StatSoft Inc., Tulsa, OK, USA).

The post-hoc power estimated on partial R^2^ according to the multivariate linear regression model adjusted for maternal weight change, breastfeeding and Cesarean section was 0.80 with α = 0.05. A *P* value of 0.002 or lower was considered as statistically significant.

## Results

### Maternal and infant gut microbiota composition

**Fig 1:**
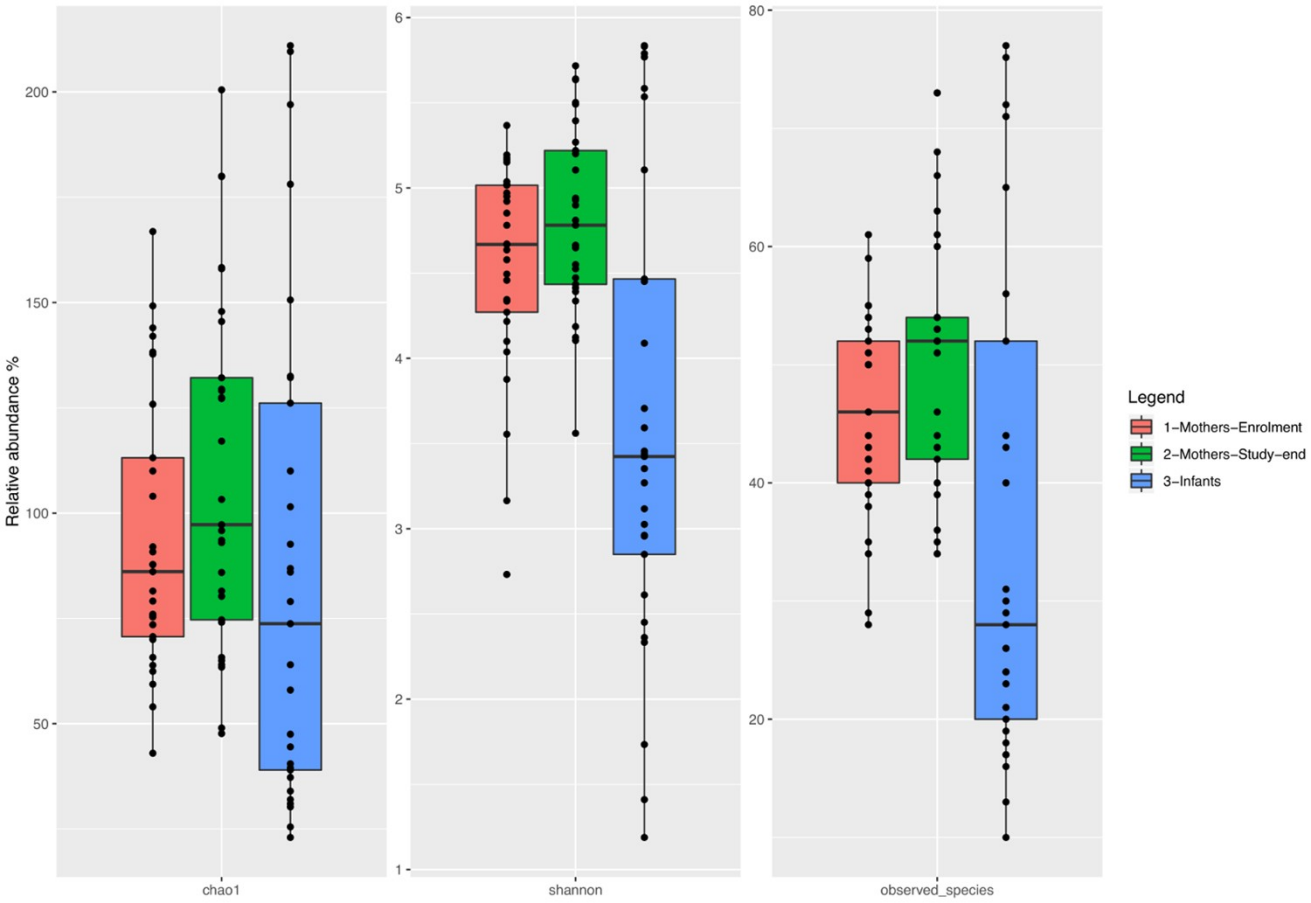
α-diversity measures of fecal microbiota. Boxplots describe α-diversity measures of fecal microbiota of GDM patients at enrolment (red bars), of GDM patients at the study end (green bars) and of their offspring (blue bars). Individual points and brackets represent the richness estimate and the theoretical standard error range, respectively.

The microbial richness (alpha diversity index) was lower in infant stools than in the corresponding maternal samples, and fewer taxa were present in the offspring (Fig 1). An increase in the rarefaction measure across pregnancy was evident in GDM patients. The number of observed species was significantly different between mother samples (both at enrolment and at the study-end) and the corresponding offspring samples (for the Chao-1 diversity index, P for paired data <0.001). The decrease in the complexity and the number of taxa observed in offspring samples was not a consequence of the lower number of infant sequences because the analysis was performed by using a rarefied down-sampling operational taxonomic unit (OTU) table.

**Fig 2:**
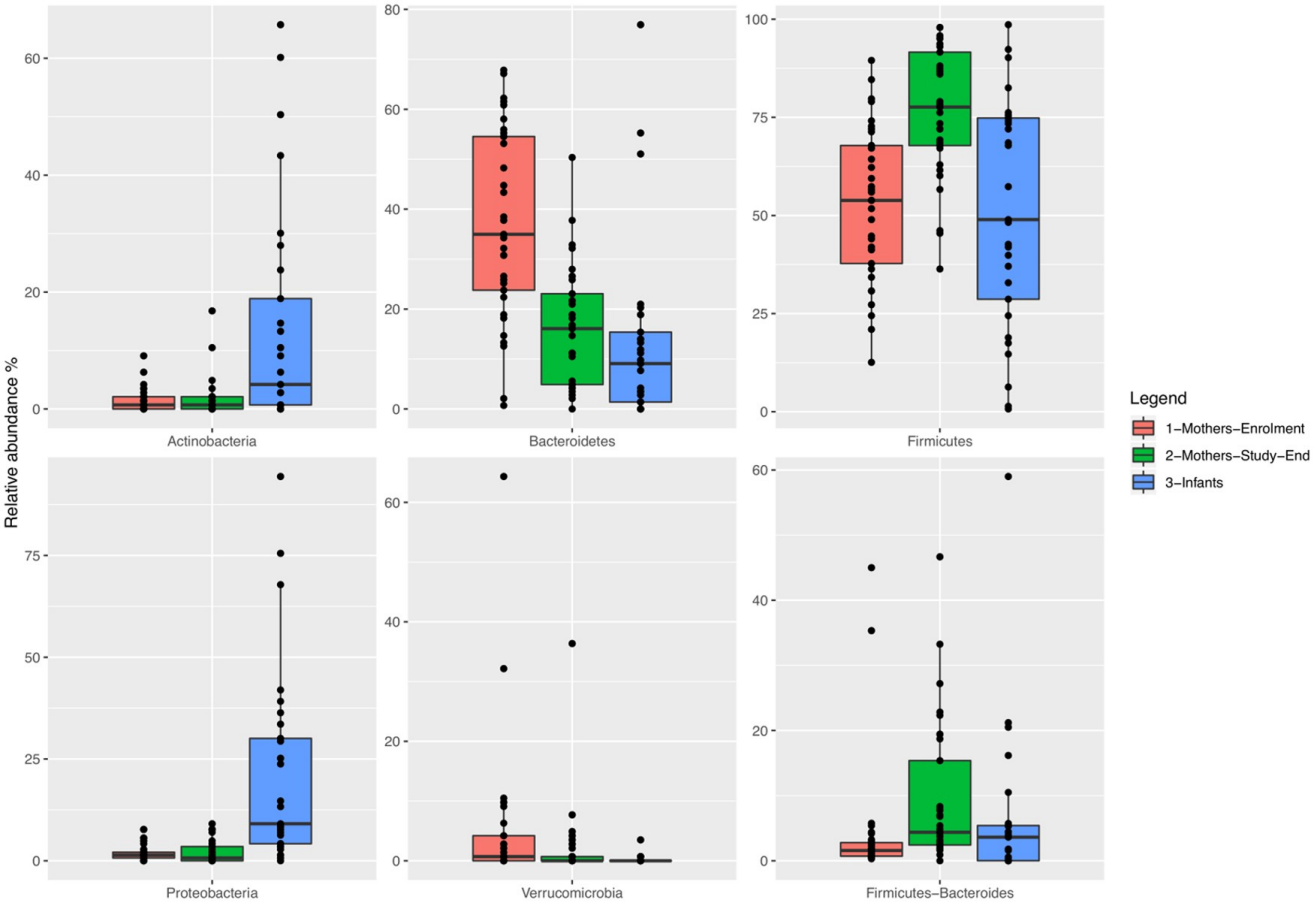
Phylum-level abundance profiles. Boxplots describe the relative abundance of phyla in GDM patients at enrolment (red bars), in GDM patients at the study end (green bars) and in the infants (blue bars).

Patients with GDM showed a predominance of Firmicutes (increasing during the gestational period) and Bacteroidetes (whose relative abundance decreased), while the infant gut microbiota was characterized by an increased relative abundance of Actinobacteria and Proteobacteria (Fig 2).

Hierarchical clustering analysis at genus level (S1 Fig) showed a separation between the infant microbiota and the mother microbiota (anosim P<0.01). Infants displayed a higher abundance of *Bifidobacterium*, *Streptococcus*, *Escherichia*, *Staphylococcus* and *Enterococcaceae* while mothers had a more complex microbiota composition (S1 Fig).

### Infant gut microbiota and maternal dietary habits and laboratory variables

**Fig 3:**
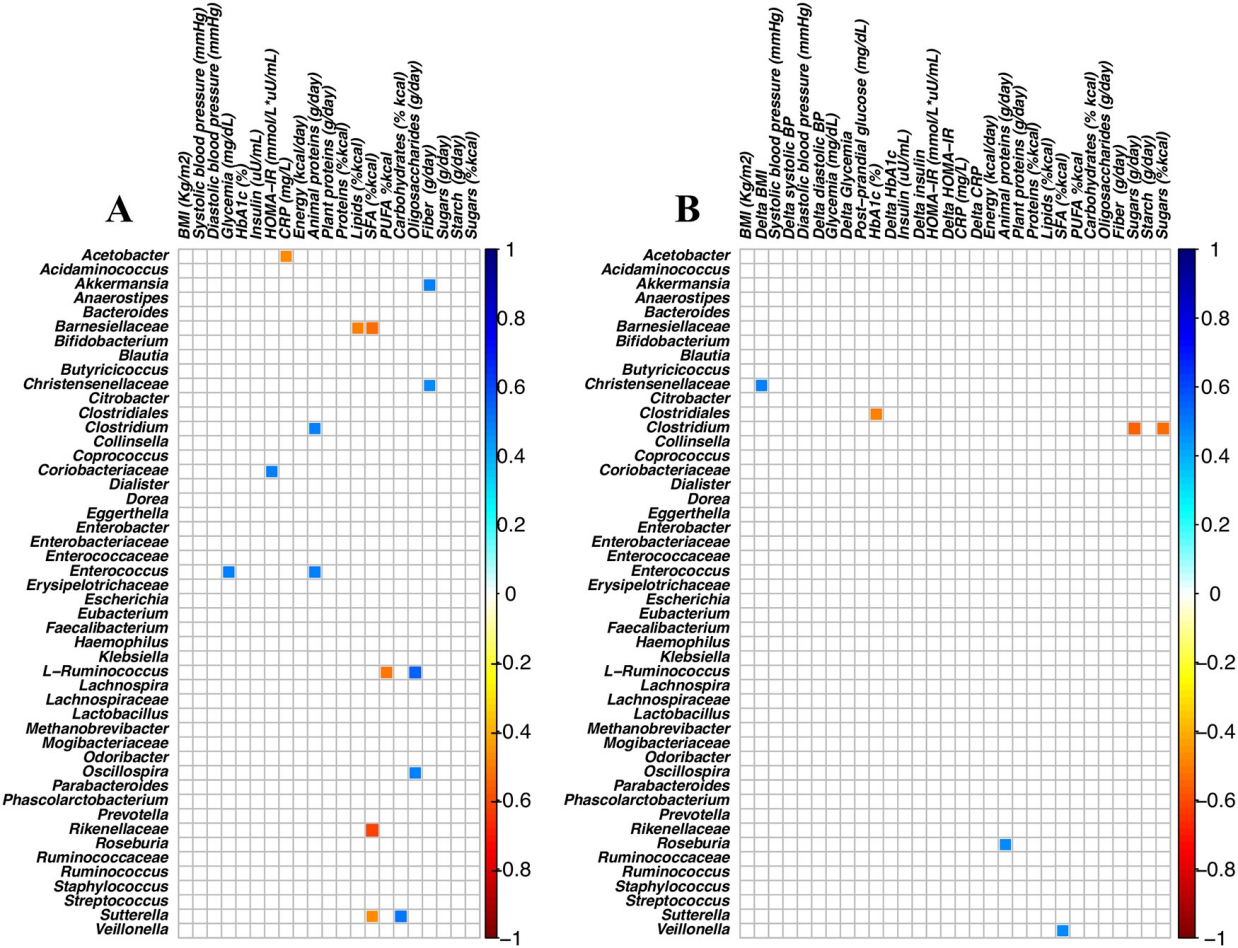
Spearman’s rank correlation of OTUs dietary information and blood variables. Spearman’s rank correlation matrix of OTUs with > 0.2% abundance in at least 10 fecal samples, dietary information and blood variables. The colors of the scale bar denote the nature of the correlation, with 1 indicating a perfectly positive correlation (dark blue) and -1 indicating a perfectly negative correlation (dark red) between the two datasets. Only correlations with P-values <0.002 are shown. Data at enrolment (plot A) or at study-end (Plot B).

No clear separation of the infant gut microbiota was observed (anosim P > 0.05) according to the maternal compliance to dietary recommendations (S2 Fig). The correlation plot between maternal nutrient intakes and infant gut microbiota showed a higher number of significant associations with maternal variables at enrolment than at the study-end (Fig 3).

Maternal oligosaccharides derived mainly from dairy products, cereals, fruit and vegetables, while saturated fatty acids (SFA) derived mainly from meat and cheese. In a multiple regression model, after adjusting for weight change, breastfeeding and Cesarean section, associations between infant *Ruminococcus* with maternal oligosaccharide (positive association) and with maternal intake of SFA (inverse association) were found, even if not reaching the defined cutoff of p-values. The inverse relationship between *Rikenellaceae* and maternal SFA intake remained statistically significant (Table 1). In the multivariate model, no significant association between infant gut microbiota and maternal dietary habits at the study-end was found.

**Table 1:** Statistically significant associations between infant microbiota composition and maternal variables by Spearman’s correlations for continuous variables (left) and multiple regression analyses (right).

	Rho	Beta	95% CI	*P*
***Dietary habits at enrolment***				
**Oligosaccharides**				
*Ruminococcus*	0.55	0.09	0.04 0.14	0.005
**Saturated fatty acids (%kcal)**				
*Rikenellaceae*	-0.61	-0.24	-0.37–0.11	**0.001**
*Ruminococcus*	-0.44	-0.76	-1.21–0.31	0.004
***Maternal laboratory variables***				
**Delta HbA1c**				
*Clostridiales*	-0.49	-0.32	-0.48–0.16	**<0.001**
Breastfeeding*				
*Bifidobacterium*	-	22.9	10.1 35.7	**0.0017**

Model adjusted for maternal weight change, breastfeeding, and Cesarean section;

*Model adjusted for maternal weight change and Cesarean section

The direct association between maternal fasting glucose at enrolment and *Enterococcus* (Rho = 0.49) was no longer confirmed in the multivariate model, while the inverse association between *Clostridiales* and maternal changes across pregnancy (delta) in glycated hemoglobin (HbA1c) levels was statistically significant (Table 1). No significant association between maternal anthropometric or metabolic variables and the gestational changes in these variables and infant gut microbiota composition was found. Two out of 29 women were treated with insulin; none received metformin. After adjusting for insulin treatment, the results of the multiple regression analyses did not change.

### Infant gut microbiota and delivery outcomes and breastfeeding

No significant associations between infant gut microbiota and delivery (Cesarean section *vs* vaginal delivery) was found.

Ten out of the 29 (34.5%) newborns were breastfed by their mothers, while 19 (65.5%) were formula-fed. We observed a non-significant reduction in microbial richness in breastfed infants when compared to infants fed with artificial milk.

**Fig 4:**
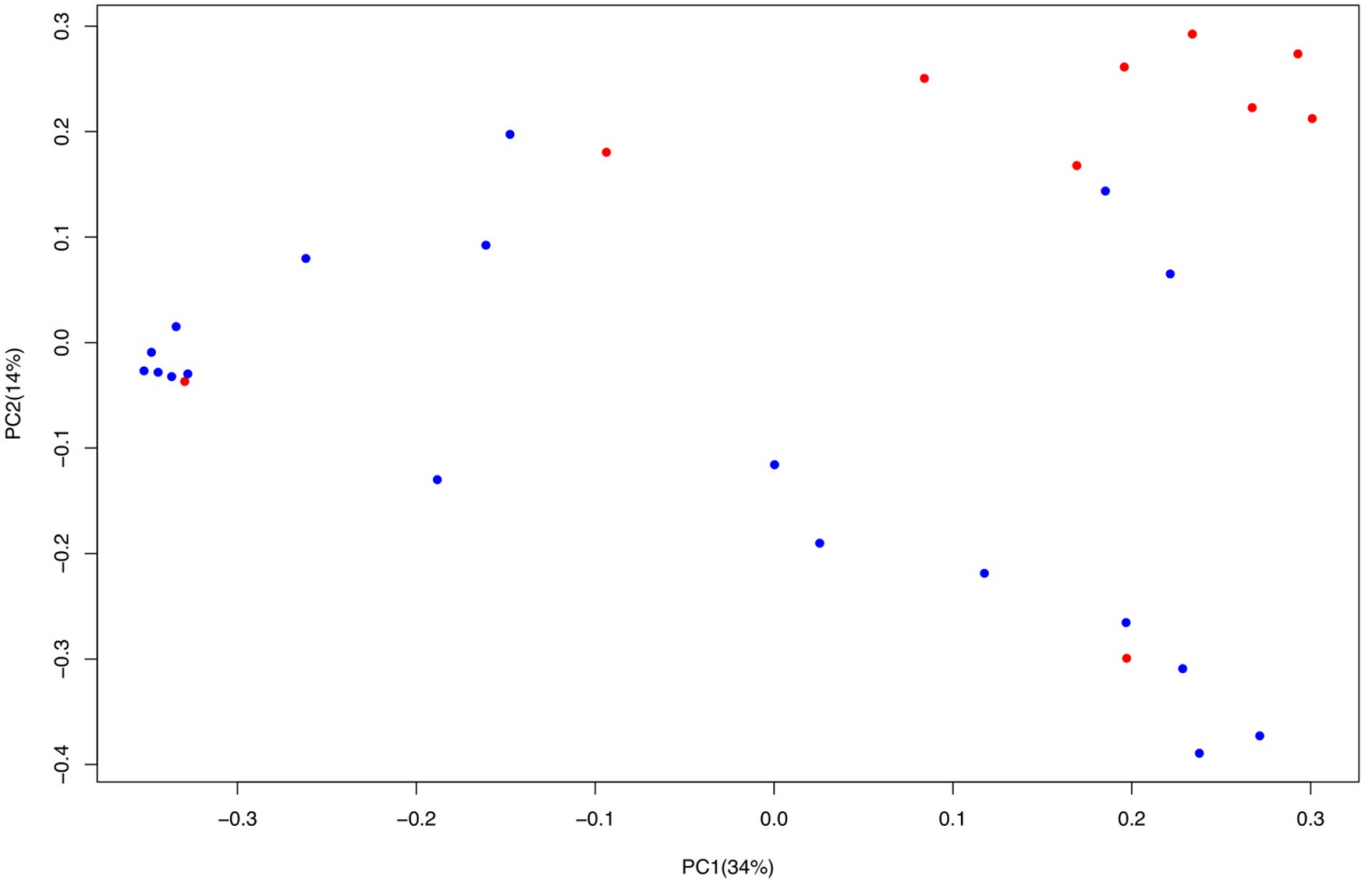
Principal coordinates analysis (PCoA) of unweighted UniFrac distances matrix for 16S rRNA gene sequence data. Infant artificially-fed (blue dots) or breast-fed (red dots).

The β–diversity calculation based on unweighted UniFrac distance matrices assessed by Principal coordinate analysis (PCoA) showed a separation of the infant microbiota only as a function of the type of feeding (breast *vs* formula) as confirmed by ANOSIM and ADONIS statistical test (P <0.001) (Fig 4).

**Fig 5:**
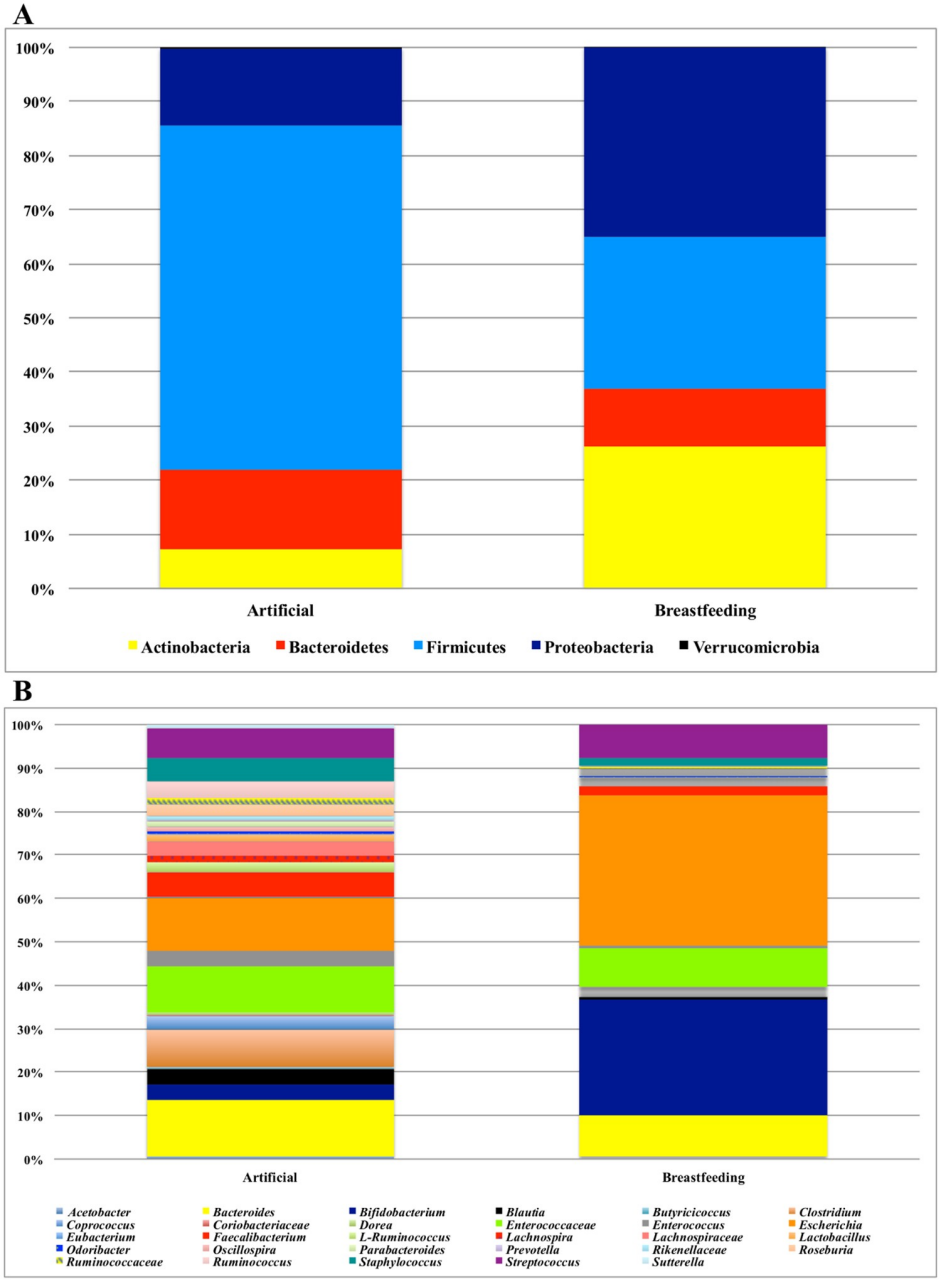
Relative phylum-level abundance profiles. Phylum-level abundance profiles of breastfed infants and artificially fed infants (Panel A); relative genus-level abundance of breastfed infants and artificially fed infants (Panel B).

At phylum level, we observed that breastfed infants showed a higher abundance of Actinobacteria and Proteobacteria, while in formula-fed infants we observed a higher proportion of Firmicutes phyla (Fig 5A). At genus level, breastfed infants displayed an increased abundance of *Escherichia* and *Bifidobacterium*, while formula-fed infants had a varied microbiota mainly composed of *Bacteroides*, *Clostridium*, *Enterococcaceae*, *Escherichia*, *Faecalibacterium*, *Staphylococcus* and *Streptococcus* (Fig 5B). In multiple regression analyses, a significant association between breastfeeding and the relative abundance of *Bifidobacterium* in the infant gut microbiota was found (β = 22.9; 95%CI = 10.1–35.7; P = 0.0017).

### Gut microbiota signature at sub-genus level

In order to explore the possible mother-to-infant gut microbiota transmission at sub-OTUs level, we carried out oligotyping on sequences of *Blautia*, *Bacteroides* and *Bifidobacterium* since these were the only genera showing a Shannon entropy index sufficient to identify all nucleotide positions that would resolve the oligotypes. We considered the dominant oligotypes shared by mothers and their offspring only to explore possible mother-to-child oligotype transmission. We then selected the oligotypes which were present in at least 5 mother-to-child pairs. We found 18 oligotypes for *Bacteroides*, 30 for *Blautia*, and 81 for *Bifidobacterium*. Only for *Bifidobacterium*, oligotypes of the offspring were taken into account, since resolving *Bifidobacterium* in mothers was not possible. A high inter-individual diversity in the *Bifidobacterium* oligotypes was observed in the offspring samples (S3 Fig). We observed that the relative abundance of the *Blautia* oligotypes increased during the pregnancy and were lower in the infants (S4A Fig). A high degree of mother-offspring concordance was found for B11 (P = 0.008) and B42 (P<0.001); both were identified as *Blautia wexlerae* by best BLASTn match. A reduction in the relative abundance of *Bacteroides* oligotypes was observed across pregnancy while few oligotypes were observed to be dominant in the offspring samples (S4B Fig).

### Comparison between gut microbiota from GDM offspring and healthy women offspring

The gut microbiota of our offspring was compared with those of 19 breastfed infants from healthy normoglycemic women. A higher α-diversity was evident in the latter (for the Chao-1 diversity index, P = 0.001). However, when comparing to those infants the 10 breastfed infants from GDM women, no significant difference in α-diversity was found. At phylum level, we observed higher abundance of Actinobacteria and Bacteroidetes in the offspring from GDM women (S5A Fig) (P<0.001). At genus level, higher abundance of *Staphylococcus*, *Ralstonia*, *Lactobacillus* and some members of *Enterobacteriaceae* were observed in the offspring from healthy women (S5B Fig).

In the comparison between the two groups of breastfed infants, the offspring of GDM women showed a significantly higher relative abundance of *Escherichia* and *Parabacteroides* (P<0.001).

## Discussion

The microbiota of infants from GDM women showed a low complexity, a high inter-individual variability, and was influenced by early maternal nutrition and breastfeeding. Intriguingly, in comparison with the offspring of healthy women, our infants showed a higher relative abundance of pro-inflammatory taxa, in particular *Escherichia* and *Parabacteroides*. A few mother-to-child oligotype transmissions were found.

The offspring gut microbiota composition substantially differs from the microbiota composition of their GDM mothers, being the former characterized by limited species-level complexity (i.e. a lower α-diversity) and a greater inter-individual variability of genus abundance (i.e. a higher β-diversity), as already reported [22,34–39]. The microbiota complexity indeed progressively increases with the infant growth, with the gradual development of a microbial community resembling the adult microbiota at the age of 3 years with a lower inter-individual variability [40–41]. Actinobacteria and Proteobacteria dominated the gut microbiota of our infants from GDM women, similarly to the infants of healthy mothers and in accordance with previous reports [36–37,42–43]. The relative abundance of these phyla has been reported to be higher in the meconium of GDM newborns compared to offspring from healthy controls [26]. *Bifidobacterium*, *Streptococcus*, *Escherichia*, *Staphylococcus* and members of the *Enterococcaceae* family displayed a higher relative abundance in the feces of our GDM infants. Accordingly, these bacteria have been detected in the feces or meconium of full-term newborns in several studies [24,34,35–37,39,44–48].

A distinct microbial composition was found in our breastfed GDM offspring when comparing them with breastfed neonates from healthy controls, consistent with literature [16,24–25]. In particular, a significantly higher relative abundance of *Escherichia* and *Parabacteroides* was found in our GDM infants. These taxa can be considered as pro-inflammatory and have been found to be higher in the meconium of babies from T2DM mothers [24]. Furthermore, *Parabacteroides* have been reported to be enriched in GDM patients when compared to healthy subjects [18]. In our previous study [17], we have observed an increased, though not statistically significant, relative abundance of *Parabacteroides* across pregnancy from enrolment to study end. Similarly, *Escherichia* is more abundant in pregnant overweight women, particularly in GDM patients [49–50] Diabetes has been considered as an inflammatory disease and, not surprisingly, the offspring microbiota may be influenced by this pregnancy pathological condition; a direct transmission from mother to child of some pro-inflammatory taxa like *Parabacteroides* or *Escherichia* might be hypothesized, even if the contribution of not analyzed environmental conditions could not be ruled out.

No differences in the Firmicutes-to-Bacteroidetes ratio, indicating an increased risk for developing obesity [51], was found neither between the breast-fed GDM infants when compared to the formula-fed GDM infants, nor between breast-fed GDM infants *vs* breast-fed infants form normoglycemic women.

The microbiota in the first days of life of our GDM offspring might be one of the contributing factors to the 2- to 8-fold increased future risk of dysmetabolic diseases of those offspring when compared to offspring of healthy women [52]. We observed that the infant gut microbiota composition is influenced by nutritional maternal habits. The offspring relative abundance of *Ruminococcus* was directly associated with the maternal intake of oligosaccharides and inversely with the maternal intake of SFA, as previously reported in infants from healthy women [53]. The *Ruminococcus* genus produces both butyrate and a bacteriocin, ruminococcin A, able to inhibit the growth of the potentially harmful *Clostridium* species, therefore potentially playing a beneficial role for the newborns [54]. Furthermore, maternal SFA intake was inversely associated with the relative abundance of *Rikenellaceae*, a butyrate-producers family [55] that has been related to favorable metabolic outcomes and a healthy gut [56–57]. These associations are remarkable and could explain the previously reported adverse impact of SFA on maternal [58] and neonatal health [59–60] even if did not reach the established significance threshold, after multiple adjustments.

Nutritional recommendations were given to our women between 24–28 weeks of gestational age, at the time of the oral glucose challenge test and GDM diagnosis [17]. Only 34.1% of our participants resulted compliant to the nutritional advice; indeed, dietary adherence and nutritional habits at the end of pregnancy did not influence the offspring microbiota. Contrarily to a few previous human studies showing that the infant gut microbiota was associated with the last-trimester maternal diet [61], we found stronger associations between infant gut bacteria composition and early maternal nutrition. Even if the mechanisms by which the maternal diet affects the offspring microbiota remain unclear, the nutritional habits of late pregnancy might have a lower impact, owing to the lower maternal-fetal time of contact and seeding possibility in a period when fetal growth is already advanced. This hypothesis needs to be confirmed by further studies. Our results indeed highlight the importance of a proper maternal nutrition, low in SFA and with a high content of fiber and prebiotic oligosaccharides, starting from early pregnancy and probably even before gestation, in order to favorably modulate the offspring microbiota.

Many other factors have been reported to influence and shape the neonatal microbiota during intrauterine life [62–63], among which those that are more frequently implicated are maternal glycemic status [24], weight gain during pregnancy [64], pre-pregnancy BMI [64–65], and antibiotic use [66]. We failed to demonstrate any associations between maternal anthropometric indexes with the offspring microbiota composition, indeed the insulin resistance and hyperglycemia of our participants, all suffering from GDM, may have obscured these relationships. Furthermore, other studies did not show relationships between maternal BMI or clinical characteristics and the infant microbiota composition [24–25].

We found an inverse association between Clostridiales and HbA1c changes across pregnancy. The Clostridiales order includes several genera and in particular *Roseburia* and *Faecalibacterium prausnitzii*, both butyrate-producing bacteria with beneficial functions, which have been found to be reduced in GDM women [15,18,22].

The microbiota of our GDM offspring did not meaningfully vary according to the way of delivery. Contrasting data are available about this topic, since a different microbial pattern [43,67–69] or no differences [8,24,36] have been reported between C-section-delivered infants and vaginally-delivered infants. It could be hypothesized that the exposure to an adverse environment in our GDM offspring could have had a major role on their gut microbiota, partly overshadowing the effects of the exposure during birth. Furthermore, the influence of delivery mode on the infant microbiota rapidly decline after delivery [40,70] and is quickly overridden by the role of lactation [39,71–72].

A clear separation of infant microbiota according to the type of feeding (breast milk vs formula) was evident, as previously reported [5,73]. In particular, our formula-fed infants showed increased microbial complexity and levels of strict anaerobes and facultative anaerobes, and higher abundance of Firmicutes, such as *Clostridium*, *Streptococcus* and *Staphylococcus*, in line with literature [2,70,74–75]. On the other hand, our breastfed infants showed a less complex microbiota with higher abundance of Proteobacteria and Actinobacteria, being the latter mainly derived from the *Bifidobacterium* genus that resulted to be strongly associated with breastfeeding, accordingly with most studies [2,75–78]. The higher abundance of *Bifidobacterium* in breast-fed infants can be explained by the presence of human milk oligosaccharides (HMOs), sugar polymers with prebiotic effects that promote the growth of specific microbial communities, including *Bifidobacterium* spp. with beneficial protective effects on the infant health [79–81]. A higher, though not significantly different abundance of Proteobacteria was evident in our breast-fed infants when compared to formula-fed infants. Literature is discordant on this topic, since either a higher abundance of Proteobacteria, in particular of *Enterobacteriaceae*, has been reported in formula-fed infants [82] or the opposite was observed, in particular a decrease in the relative abundance of Proteobacteria in non-breast-fed *vs* breast-fed infants [83]. Differences in breastmilk microbiota and HMOs content, maternal diet and type of formula milk used across populations might be responsible for these heterogeneous results.

A possible vertical mother-to-child transmission of maternal gut bacteria has already been reported, even if, to date, certainty about the way of intrauterine microbial acquisition is lacking [37,84–85]. Besides breastfeeding and vaginal microbiota, also placenta and amniotic fluid have been reported to be a vehicle for this transmission [22,86]. By using the oligotype pipeline, the transmission of different sub-OTUs from our mothers to their infants was observed in line with data of a recent metagenomic study confirming that mother’s dominant strains were transmitted to their children [42]. In particular, we have found a few *Bacteroides* and *Blautia* oligotypes significantly shared by the GDM mothers and their offspring, suggesting a maternal microbial imprinting. On the other hand, the infant *Bifidobacterium* oligotypes of the newborns were not present in the feces of the GDM women, and this directs towards a post-natal acquisition. Indeed, the infant *Bifidobacteria* mostly derive from breast-feeding, being either isolated from human milk and vertically transmitted [86–87] or induced by the milk prebiotic HMO [88]. The *Bifidobacterium* oligotypes were highly different among infants but only one third of them was breastfed and the human milk has a heterogeneous composition and contains variable amounts of glycans and bifidogenic oligosaccharides [72,89].

In conclusion, the gut microbiota changes might be one of the conditions determining the increased metabolic risk of the GDM offspring with both pre and postnatal effects, since in utero exposure to maternal nutrition and dysmetabolic conditions, and post-natal breastfeeding can both modulate the offspring microbial community.

Several limitations of the present study should be recognized. The sample size was low, the standard errors were wide, and we could analyze the fecal samples of only 70.7% of the offspring of the initially enrolled women. Therefore, we might not have detected modest differences in bacterial composition. Many associations that we found to be not statistically significant, could have become so if we had analyzed a larger sample. However, the post-hoc power was 0.80 with α = 0.05; furthermore, we have set-up a lower p-value cut-off value (p<0.002) as statistically significant. In doing so, we have considered only the strongest associations; however, we cannot exclude the possibility that a type II error could have occurred.

Infant fecal samples were collected between the 3^rd^ and the 5^th^ days of life. We cannot exclude that the difference of a few days could have influenced the results due to the high instability of the infant microbiota.

## Conclusions

Early maternal eating habits and breastfeeding may have a significant influence on the fetal gut microbiota composition of infants from GDM women. These results are worthy to be replicated in larger studies owing to their potential practical implications on the health of future generations.

## Supporting information

S1 Fig Heat-plot of the abundances of OTUs at genus level. Heatplot showing OTUs of GDM patients at enrolment (red bars), study end (green bars) and their offspring (blue bars). Rows and columns are clustered by means of Ward linkage hierarchical clustering. The intensity of the colors represents the degree of correlation between the samples and OTUs as measured by Spearman’s correlations.
(TIF)

S2 Fig Principal Component Analysis (PCA) based on OTUs relative abundance of infant gut microbiota according to the maternal compliance to dietary recommendations. Samples are color-coded according to compliance to dietary recommendations (red) or non-compliance to dietary recommendations (blue).
(TIF)

S3 Fig Distribution of representative *Bifidobacterium* oligotypes in the offspring samples. Inner black bars indicate the presence of an oligotype in a given sample.
(TIF)

S4 Fig Distribution in representative *Blautia* (plot A) and *Bacteroides* (plot B) oligotypes. Plot showing the sequence distribution in GDM patients at enrolment (red bars), study end (green bars) and their offspring (blue bars). Inner bars indicate the presence of an oligotype in a given sample. Outer circle, if colored, denotes oligotype abundance with high degree of mother-offspring concordance.
(TIF)

S5 Fig Distribution of OTUs between offspring from healthy and GDM women. Plot shows the relative phyla (panel A) and genus (panel B) abundance in the offspring from healthy and GDM women.
(TIF)

S1 Table Enrolment characteristics and pregnancy outcomes of the GDM women. (DOCX)
