# Supplementary figures and images for: The microbiota composition of the offspring of patients with gestational diabetes mellitus (GDM)
Source: PLoS One. 2019 Dec 16;14(12):e0226545. doi: 10.1371/journal.pone.0226545 (PMC6913919; doi:10.1371/journal.pone.0226545)

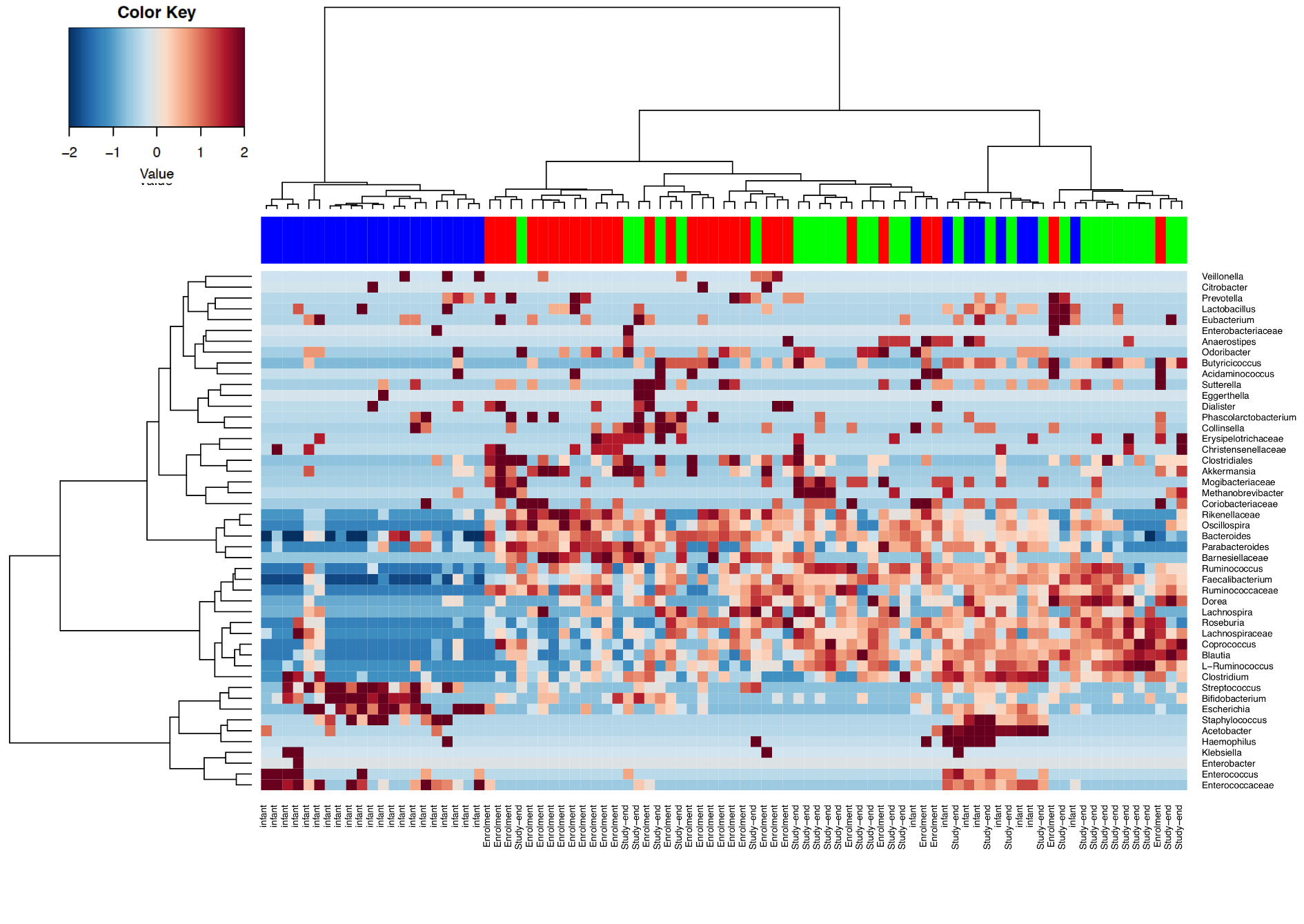

Supplement: S1 Fig — Heatplot showing OTUs of GDM patients at enrolment (red bars), study end (green bars) and their offspring (blue bars). Rows and columns are clustered by means of Ward linkage hierarchical clustering. The intensity of the colors represents the degree of correlation between the samples and OTUs as measured by Spearman’s correlations. (TIF) [file pone.0226545.s001.tif]

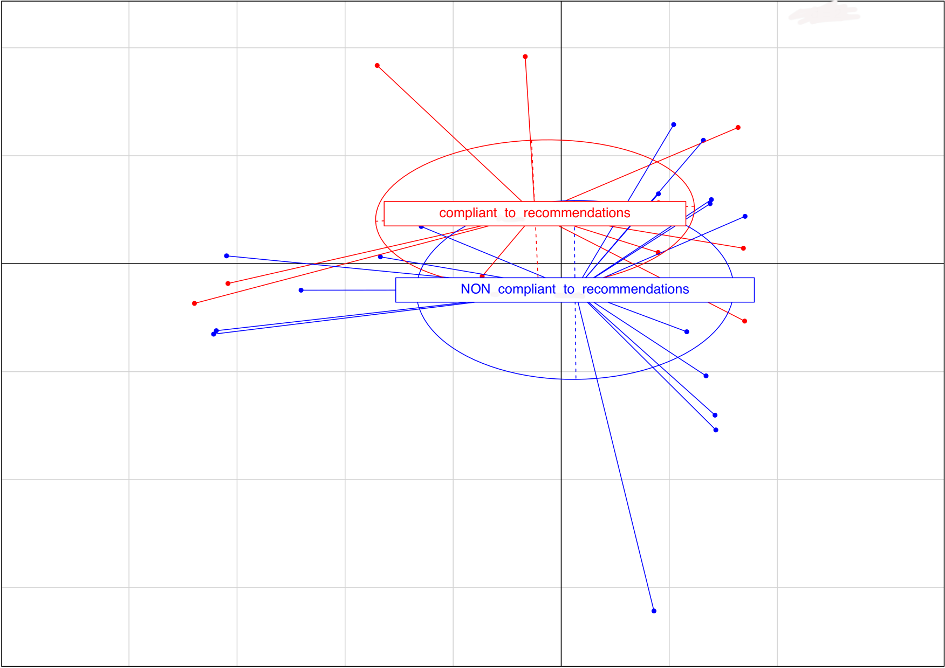

Supplement: S2 Fig — Samples are color-coded according to compliance to dietary recommendations (red) or non-compliance to dietary recommendations (blue). (TIF) [file pone.0226545.s002.tif]

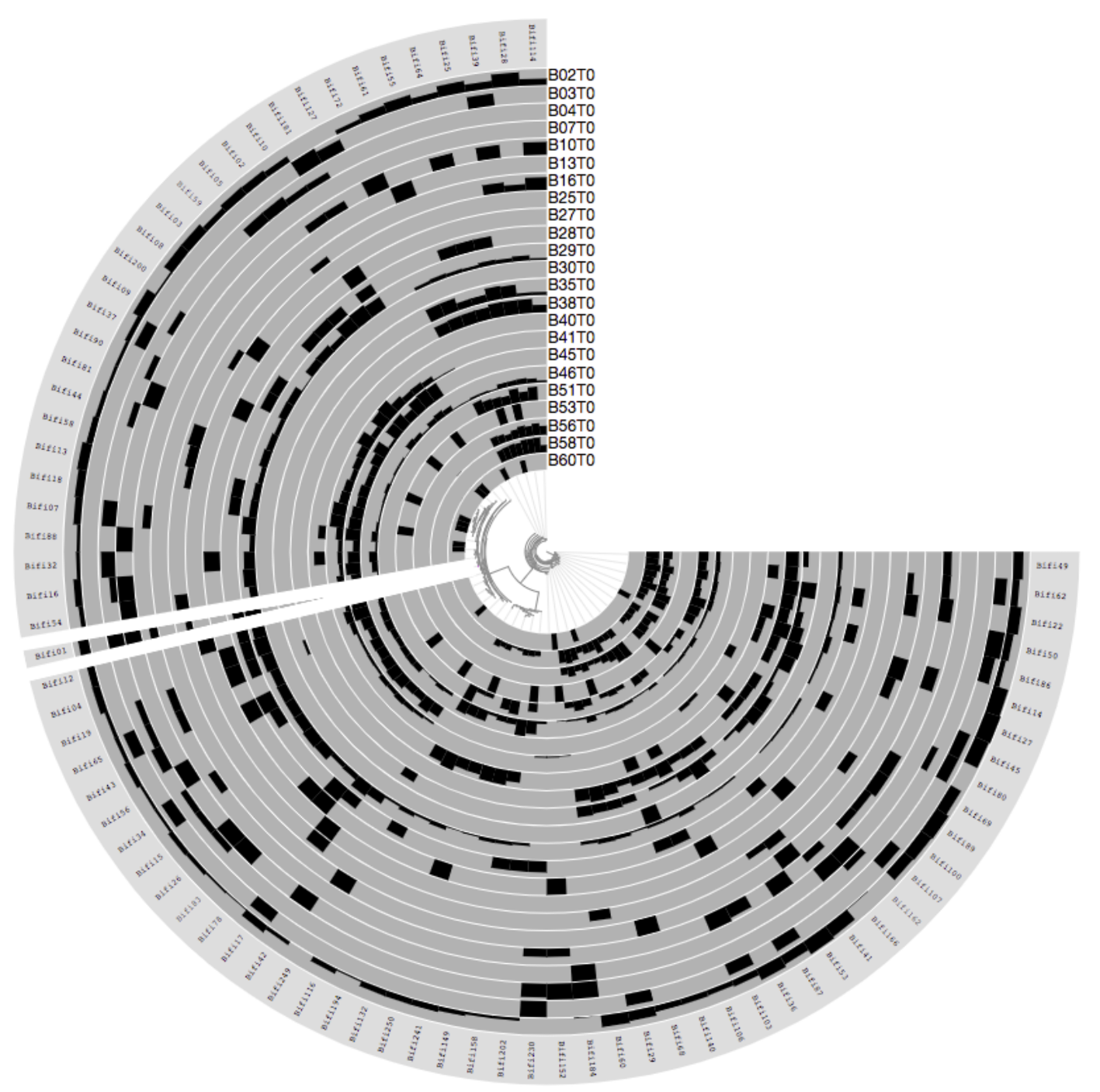

Supplement: S3 Fig — Inner black bars indicate the presence of an oligotype in a given sample. (TIF) [file pone.0226545.s003.tif]

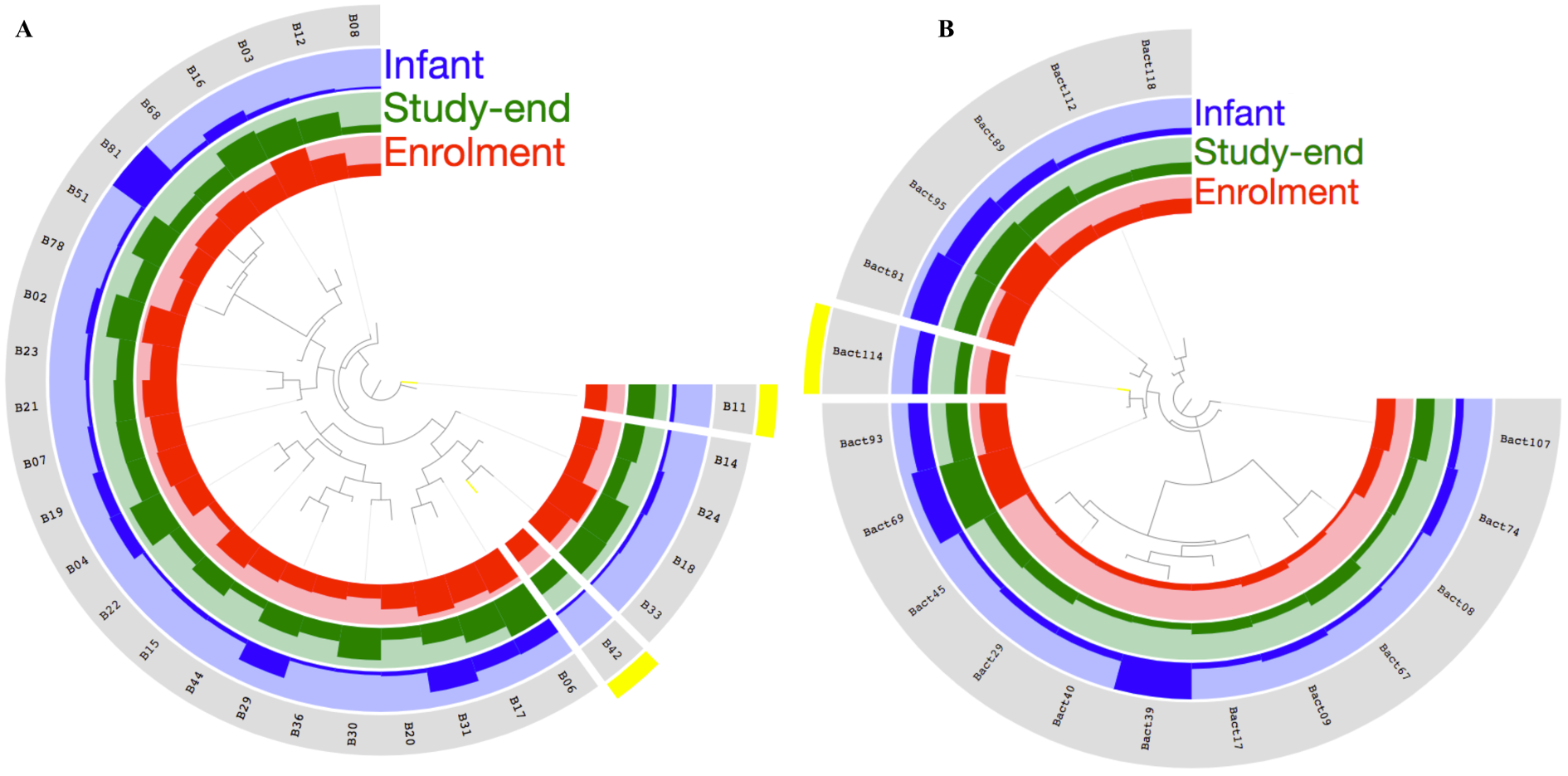

Supplement: S4 Fig — Plot showing the sequence distribution in GDM patients at enrolment (red bars), study end (green bars) and their offspring (blue bars). Inner bars indicate the presence of an oligotype in a given sample. Outer circle, if colored, denotes oligotype abundance with high degree of mother-offspring concordance. (TIF) [file pone.0226545.s004.tif]

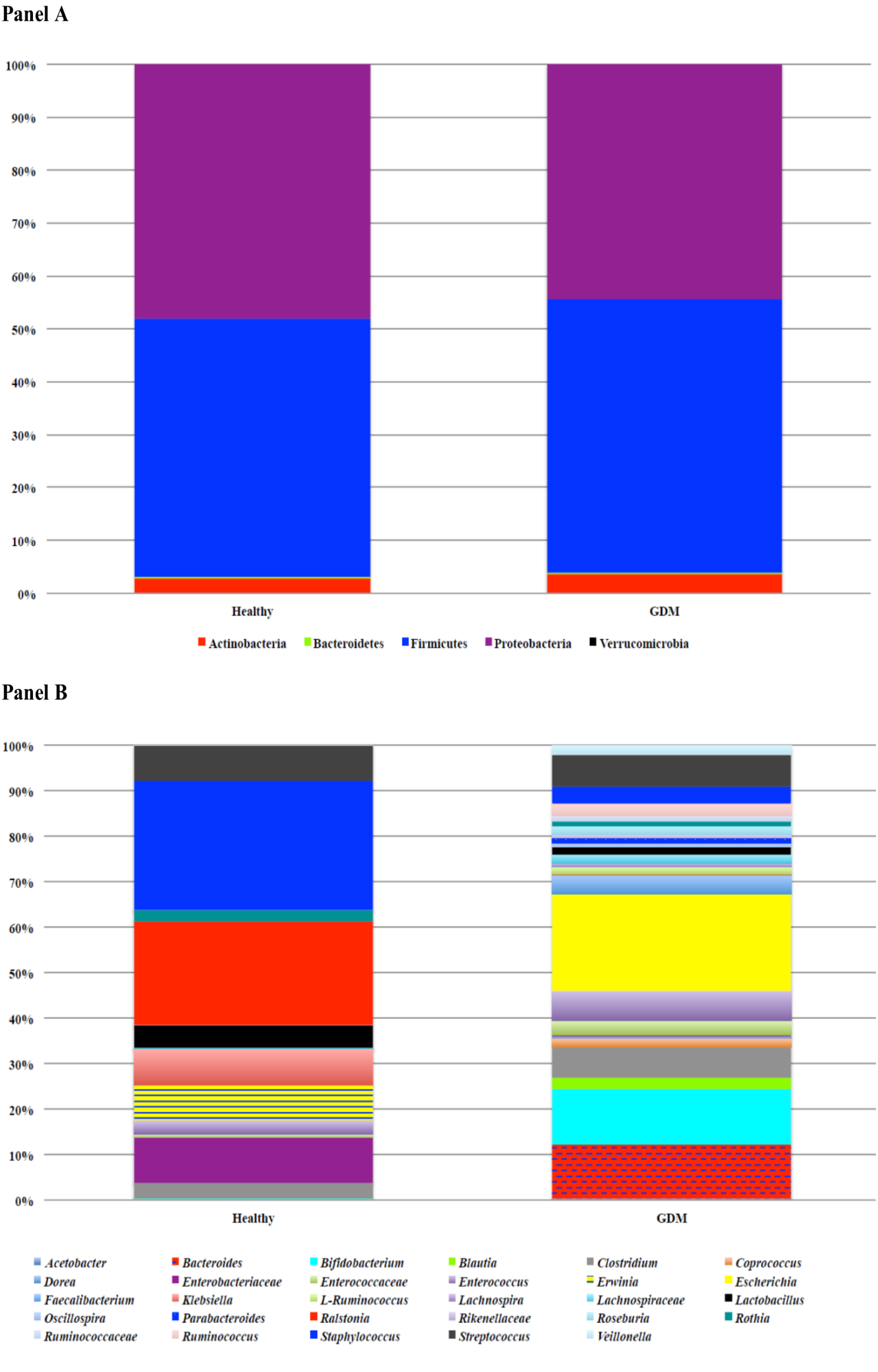

Supplement: S5 Fig — Plot shows the relative phyla (panel A) and genus (panel B) abundance in the offspring from healthy and GDM women. (TIF) [file pone.0226545.s005.tif]
