# Supplementary material for: The microbiota composition of the offspring of patients with gestational diabetes mellitus (GDM)
Source: PLoS One. 2019 Dec 16;14(12):e0226545. doi: 10.1371/journal.pone.0226545 (PMC6913919; doi:10.1371/journal.pone.0226545)
Supplement: S1 Table — (DOCX) [file pone.0226545.s006.docx]

**Supplementary Table 1. Enrolment characteristics and pregnancy outcomes of the GDM women**

| Number | 29 |
| --- | --- |
| Age (years) | 37.1±4.5 |
| Pre-pregnancy weight (kg) | 71.2±15.1 |
| Pre-pregnancy BMI (kg/m^2^) | 26.4±5.9 |
| Nulliparous (%) | 58.6 |
| Weight (kg) | 77.0±13.4 |
| BMI (kg/m^2^) | 28.5±5.1 |
| Fasting glucose (mg/dL) | 95.9±13.7 |
| Systolic blood pressure (mmHg) | 109.0±11.7 |
| Diastolic blood pressure (mmHg) | 73.2±7.9 |
| Glycated hemoglobin (%) | 4.7±0.8 |
| Fasting insulin (µU/mL) | 10.1 (8.2) |
| HOMA-IR (mmol/L*µU/mL) | 2.3 (1.6) |
| Total cholesterol (mg/dL) | 237.7±30.2 |
| HDL-cholesterol (mg/dL) | 66.3±11.3 |
| Triglycerides (mg/dL) | 172.0±52.1 |
| C-reactive protein (mg/L) | 3.8 (4.4) |
| *Dietary intakes* |  |
| Energy (kcal) | 1599.7±209.4 |
| Carbohydrates (% total kcal) | 44.6±7.3 |
| Sugars (% total kcal) | 8.0±3.7 |
| Sugars (g/day) | 31.9±15.0 |
| Oligosaccharides (g/day) | 39.4±19.7 |
| Starch (g/day) | 108.5±27.6 |
| Fiber (g/day) | 15.0±4.4 |
| Proteins (% total kcal) | 15.6±2.5 |
| Total fats (% total kcal) | 42.0±5.6 |
| Saturated fatty acids (% total kcal) | 11.1±2.2 |
| Polyunsaturated fatty acids (% kcal) | 4.5±1.1 |
| *Pregnancy outcomes* |  |
| Insulin treatment (%) | 6.9 |
| Cesarean section (%) | 27.6 |
| Gestational age at delivery (weeks) | 39.1±1.3 |
| Large for gestational age newborns (%) | 6.9 |
| Male newborns (%) | 51.7 |

BMI=body mass index, HOMA-IR=Homeostasis Model Assessment-Insulin Resistance, HDL=high density lipoprotein, Values are expressed as mean ± standard deviation or median (interquartile range)
